# Evaluating the Effect of Climate on Viral Respiratory Diseases Among Children Using AI

**DOI:** 10.3390/jcm13237474

**Published:** 2024-12-09

**Authors:** Mikhail I. Krivonosov, Ekaterina Pazukhina, Alexey Zaikin, Francesca Viozzi, Ilaria Lazzareschi, Lavinia Manca, Annamaria Caci, Rosaria Santangelo, Maurizio Sanguinetti, Francesca Raffaelli, Barbara Fiori, Giuseppe Zampino, Piero Valentini, Daniel Munblit, Oleg Blyuss, Danilo Buonsenso

**Affiliations:** 1 Research Center in Artificial Intelligence, Lobachevsky State University, 603022 Nizhny Novgorod, Russia; 2 Wolfson Institute of Population Health, Queen Mary University of London, London EC1M 6BQ, UK; 3 Institute for Cognitive Neuroscience, University Higher School of Economics, 109028 Moscow, Russia; 4 Department of Mathematics and Women’s Cancer, University College London, London WC1E 6BT, UK; 5 Medicine and Surgery, Università Cattolica del Sacro Cuore, 00168 Rome, Italy; 6 Department of Woman and Child Health, Fondazione Policlinico Universitario A. Gemelli IRCCS, 00168 Rome, Italy; 7 Dipartimento di Scienze di Laboratorio e Infettivologiche, Fondazione Policlinico Universitario A. Gemelli IRCCS, 00168 Rome, Italy; 8 Dipartimento di Scienze Biotecnologiche di Base, Cliniche Intensivologiche e Perioperatorie-Sezione di Microbiologia, Università Cattolica del Sacro Cuore, 00168 Rome, Italy; 9 Care for Long Term Conditions Division, Florence Nightingale Faculty of Nursing, Midwifery and Palliative Care, King’s College London, London SE1 8WA, UK; 10 Department of Paediatrics and Paediatric Infectious Diseases, Institute of Child’s Health, Sechenov First Moscow State Medical University (Sechenov University), 119991 Moscow, Russia; 11 Centro di Salute Globale, Università Cattolica del Sacro Cuore, 00168 Rome, Italy

**Keywords:** pediatric respiratory infections, climate variables, machine learning predictions

## Abstract

**Background**: Respiratory viral infections (RVIs) exhibit seasonal patterns influenced by biological, ecological, and climatic factors. Weather variables such as temperature, humidity, and wind impact the transmission of droplet-borne viruses, potentially affecting disease severity. However, the role of climate in predicting complications in pediatric RVIs remains unclear, particularly in the context of climate-change-driven extreme weather events. **Methods**: This retrospective cohort study analyzed 1610 hospitalization records of children (0–18 years) with lower respiratory tract infections in Rome, Italy, between 2018 and 2023. Viral pathogens were identified using nasopharyngeal molecular testing, and weather data from the week preceding hospitalization were collected. Several machine learning models were tested, including logistic regression and random forest, comparing the baseline (demographic and clinical) models with those including climate variables. **Results**: Logistic regression showed a slight improvement in predicting severe RVIs with the inclusion of weather variables, with accuracy increasing from 0.785 to 0.793. Average temperature, dew point, and humidity emerged as significant contributors. Other algorithms did not demonstrate similar improvements. **Conclusions**: Climate variables can enhance logistic regression models’ ability to predict RVI severity, but their inconsistent impact across algorithms highlights challenges in integrating environmental data into clinical predictions. Further research is needed to refine these models for use in reliable healthcare applications.

## 1. Introduction

Respiratory viral infections (RVIs), such as those caused by influenza viruses, respiratory syncytial virus, and coronaviruses, are among the leading causes of morbidity and mortality globally, particularly in vulnerable populations such as the elderly and children. Severity of infections varies substantially across different age groups, with children often serving as major reservoirs and amplifiers of community transmission. Multiple biological, ecological, and socioeconomic factors contribute to the spread of respiratory infectious diseases. RVIs demonstrate strong seasonality, with multiple underlying mechanisms contributing to it [1,2,3,4]. On the population side of this issue, these factors include variation in immune response and changes in behavioral patterns. For example, children spend more time indoors during cold weather; therefore, spending more time indoors with reduced ventilation could lead to infection outbreaks. On the climate side, temperature, humidity, precipitation, sunlight, and wind speed have been identified as meaningful factors for RVI spread [5]. For instance, excessive humidity can lead to the growth of mold, which is associated with bronchitis, coughing, wheezing, chest infections, shortness of breath, rhinitis, and allergic reactions among children [6]. Humidity, wind, and temperature affect droplet sedimentation and evaporation—the key pathways of transmission of droplet-borne viruses from infected to uninfected people [7,8,9,10]. Heatwaves cause increased air pollution, which deteriorates respiratory health [11,12]. However, due to climate change, the waves of RVIs are becoming more and more dependent on abnormal weather fluctuations rather than the seasonal ones. Extreme weather events provoke vector-borne disease outbreaks by affecting pathogens, vectors, and hosts [5]. In addition, individual indoor climate conditions significantly modify the patterns formed by outdoor climate.

There is moderate evidence that climate conditions are related to severity of RVI among children [13,14]. The goal of this study is to find out whether the weather conditions before hospitalization can be used to predict the complications of respiratory infections in hospitalized children.

## 2. Materials and Methods

Our retrospective cohort study included children 0–18 years old hospitalized with LRTI (lower respiratory tract infection) symptoms at the Fondazione Policlinico Universitario A. Gemelli IRCCS of Rome, Italy, between January 2018 and December 2023. The viral pathogens were detected in nasopharyngeal molecules via polymerase chain reaction tests upon hospitalization. More details about this study can be found in [15].

The primary outcome of interest was RVI severity, defined as any of the following treatments during hospitalization: oxygen therapy, low-flow oxygen delivery, HFNC (high-flow nasal cannula), CPAP (constant positive airway pressure), and VM (mechanical ventilation). We included the following variables, collected retrospectively: age, gender, ethnicity, heart disease, tumor or immune deficit, respiratory disease, neuromuscular disease, gastrointestinal disease, and positive tests determined via nasal swab upon hospitalization for a set of viruses, namely, influenza, adenovirus, enterovirus, parainfluenza, metapneumovirus, bocavirus, rhinovirus, coronavirus, SARS-CoV, and isolated virus. In addition, we included the following weather characteristics occurring during the week preceding hospitalization: average temperature, maximal average temperature, minimal average temperature, average humidity, average wind speed, maximal wind speed, average pressure, rain, and fog.

To evaluate the predictive potential of the weather covariates, we used multiple AI approaches: logistic regression, decision trees, random forests, support vector machine, and a neural network with one hidden layer. We compared two model specifications: baseline (demographic characteristics, comorbidities, and viral infection types) and extended (baseline and weather characteristics). We preselected variables before including them in the models, using a criterion *p*-value less than 0.2. We conducted analysis in R version 4.4.1 using the packages MASS, caret, e1071, randomForest, xgboost, and neuralnet. Continuous variables were standardized. We divided the dataset into training and testing subsets using a 70:30 ratio. To interpret the results of the best predictive model, we used SHAP approach [16] and the corresponding shapr package for R. Robustness of the best model was evaluated using the ProjectedGradientDescent Attack approach from the Adversarial-Robustness-Toolbox for Python version 3.9 [17].

## 3. Results

Of the 1643 records about hospitalization, 1610 contained complete data, with 134 children having multiple hospitalizations. Patients with severe cases were slightly younger and had more respiratory comorbidities than the patients with non-severe cases. Severe cases were half as frequent as non-severe cases (Table 1). Non-severe-case children were almost twice as old as the children with severe infections. The most common preconditions were neuromuscular diseases in non-severe cases (6.3%) and respiratory diseases in severe cases (14.0%).

Based on the *p*-value criteria, we excluded ethnicity, heart disease, other diseases, parainfluenza, coronavirus, average wind, average pressure above sea level, and fog. Other variables were included in the models.

The results of the predictions are presented in Table 2. Overall, adding weather characteristics did not significantly improve the quality of the prediction of severity among hospitalized children. We improved the quality of prediction in the logistic regression, whereas the other algorithms either made worse predictions (random forest and neural network) or automatically dropped the climate variables due to considering them irrelevant (decision trees, SVM, and XGBoost). The best model, logistic regression, had an accuracy of 0.785 (0.745–0.821) with the basic set of covariates and 0.793 (0.754–0.828) when adding climate variables.

**Figure 1:**
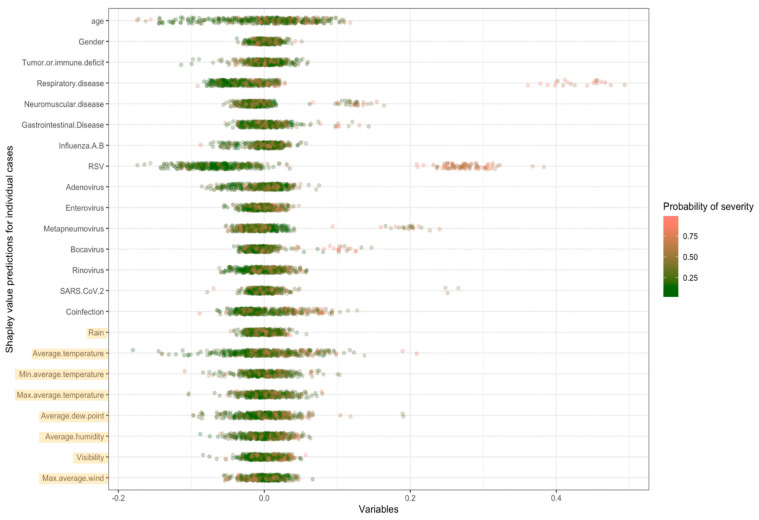
Shapley values for all predictors arranged by individual cases.

**Figure 2:**
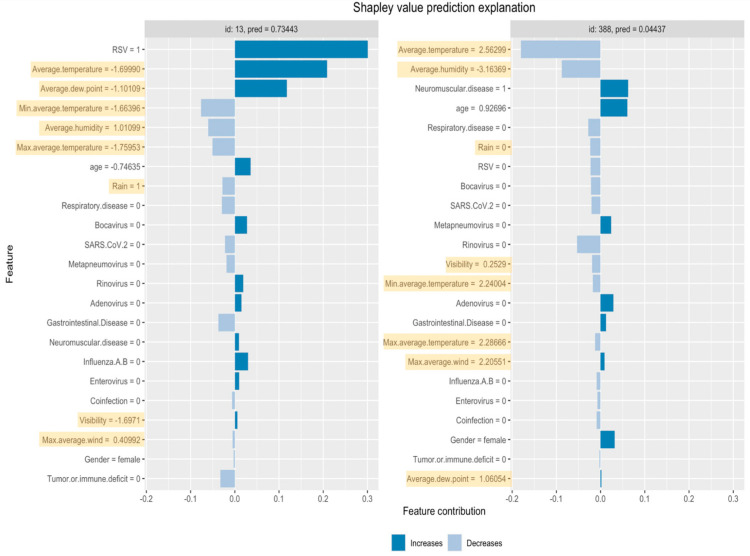
Shapley values with the largest positive and the largest negative contribution of average temperature variable.

Variable importance analysis showed that for logistic regression, three climate variables (average temperature, average dew point, and average humidity) were among the top 10 most important variables (Table 3). Average temperature made a relatively large positive contribution to several severe cases and a relatively large negative contribution to multiple non-severe cases (Figure 1). In some cases, both with high and low predicted probabilities of severity, climate variables were among the top contributors to the predicted probability of the outcome (Figure 2).

The climate variables slightly improved the performance of the logistic models for both the original and adversarial datasets, proving the robustness of our results (Table 4).

In evaluating the predictive models, the variability in performance across algorithms highlights the complexity of ensuring reliability in AI-driven healthcare applications. Logistic regression showed improvements with the inclusion of climate variables, while other models such as random forest and neural networks demonstrated poorer predictive performance. Additionally, decision trees, SVM, and XGBoost automatically excluded climate variables, flagging concerns about their roles in prediction consistency. These findings underscore the challenges of aligning predictive algorithms with the principles of Trustworthy AI, which requires models to not only deliver accurate predictions but also maintain consistent reliability when integrating diverse data sources, like climate information, into healthcare predictions.

## 4. Discussion

Our study has shown that information about climate can improve the quality of the prediction of the severity of RVI among hospitalized children, which is in line with the previous research findings about the relationship between climate and the risk of viral infections. Average temperature, dew point, and humidity contributed to the prediction of severity of LRTI among hospitalized children. However, several AI approaches did not use this information effectively, which led to predictions with either the same or even worse quality. According to the SHAP approach of evaluating the contribution of features to the predicted outcome, in some individual cases, the climate variables were among the top contributors to the prediction. Further research is needed to determine whether the selected AI approaches and model specifications are adequate for the current research question.

These findings raise important considerations for Trustworthy AI in healthcare, emphasizing that beyond performance metrics, models must also be evaluated for their reliability, fairness, and interpretability. In this context, the slight gains in logistic regression suggest that climate variables may hold some relevance, but the inconsistency across algorithms points to the need for careful scrutiny when introducing external data sources.

Trustworthy AI mandates not only transparency in how models function but also the assurance that predictions are stable and explainable, especially when these models influence clinical decisions. This study underlines the importance of conducting thorough model validation and ensuring that any integration of additional data, such as environmental factors, does not inadvertently compromise model integrity. Going forward, it is crucial to refine the application of AI in healthcare to ensure that models are both scientifically rigorous and ethically aligned with patient-centered care.

Our study has several limitations. The data included only hospitalized children, which could lead to biases towards more severe cases and reflect the varying hospitalization criteria across 5 years, including the period of the COVID-19 pandemic. Moreover, only hospitalizations from the city of Rome were included, limiting the possible range of climate variables to a single region. Still, even with the limited variation in climate variables, we can see that some models were improved after including these variables as features. Another limitation is that climate can impact not just the severity of viral infections but also the absolute number of infected or hospitalized patients. We have chosen this angle for our study because we wanted to evaluate the potential burden of climate change on public health systems arising from the severity of some infections.

## Figures and Tables

**Table 1:** Characteristics of the patients and the weather conditions before their hospitalizations.

Characteristic	Non-Severe	Severe	*p*-Value ^2^
	**N = 1108 ^1^**	**N = 502 ^1^**	
Gender			0.089
female	475 (43%)	238 (47%)	
male	633 (57%)	264 (53%)	
Age	2.4 (0.8, 5.3)	1.1 (0.2, 3.4)	<0.001
Ethnicity			0.500
Italian	899 (81%)	415 (83%)	
Other	209 (19%)	87 (17%)	
**Comorbidities**			
Heart disease	23 (2.1%)	13 (2.6%)	0.500
Tumor or immune deficit	25 (2.3%)	3 (0.6%)	0.018
Respiratory disease	23 (2.1%)	71 (14%)	<0.001
Neuromuscular disease	70 (6.3%)	57 (11%)	<0.001
Gastrointestinal Disease	15 (1.4%)	15 (3.0%)	0.025
Other diseases	165 (15%)	86 (17%)	0.300
**Viruses**			
Influenza A/B	111 (10%)	31 (6.2%)	0.012
RSV	101 (9.1%)	191 (38%)	<0.001
Adenovirus	226 (20%)	58 (12%)	<0.001
Enterovirus	320 (29%)	173 (34%)	0.024
Parainfluenza	56 (5.1%)	29 (5.8%)	0.500
Metapneumovirus	32 (2.9%)	31 (6.2%)	0.002
Bocavirus	47 (4.2%)	39 (7.8%)	0.004
Rhinovirus	410 (37%)	211 (42%)	0.055
Coronavirus	52 (4.7%)	19 (3.8%)	0.400
SARS-CoV-2	7 (0.6%)	6 (1.2%)	0.200
Coinfection	384 (35%)	236 (47%)	<0.001
**Climate variables**			
Average temperature	13.7 (11.3, 20.4)	13.0 (10.3, 18.0)	<0.001
Min average temperature	9.5 (7.0, 15.0)	9.3 (6.0, 13.6)	0.005
Max average temperature	18 (15, 25)	17 (15, 22)	<0.001
Average dew point	9.3 (6.4, 13.1)	9.1 (5.9, 12.3)	0.090
Average humidity	70 (64, 77)	74 (67, 79)	<0.001
Visibility	19.30 (18.60, 20.00)	19.10 (18.30, 19.70)	0.006
Average wind	10.10 (8.70, 12.00)	10.10 (8.60, 12.10)	0.800
Max average wind	19.8 (17.4, 22.7)	19.4 (16.7, 22.4)	0.059
Average pressure above sea level	1015.0 (1011.7, 1018.0)	1,015.0 (1011.0, 1018.9)	>0.9
Rain	779 (70%)	389 (77%)	0.003
Fog	212 (19%)	103 (21%)	0.500

^1^ N (%); median (Q1, Q3). ^2^ Pearson’s chi-squared test for categorical variables with any expected cell count above or equal to 5; Wilcoxon rank sum test for continuous variables with two levels; Fisher’s exact test for categorical variables with any expected cell count below 5.

**Table 2:** Predictive performance of models with and without climate variables for severity of viral respiratory infections in hospitalized children.

Models	Variables	Accuracy (95% CI)	AUC ^1^	Sensitivity	Specificity	PPV ^1^	NPV ^1^	Precision	Recall	F1
Logistic	Basic	0.785 (0.745–0.821)	0.703	0.824	0.648	0.89	0.515	0.890	0.824	0.856
	Basic + Climate	0.793 (0.754–0.828)	0.720	0.835	0.658	0.888	0.551	0.888	0.835	0.86
Decision tree	Basic	0.774 (0.734–0.811)	0.671	0.804	0.648	0.908	0.434	0.908	0.804	0.853
	Basic + Climate	0.774 (0.734–0.811)	0.671	0.804	0.648	0.908	0.434	0.908	0.804	0.853
Support Vector Machines	Basic	0.783 (0.743–0.819)	0.694	0.818	0.65	0.896	0.493	0.896	0.818	0.856
	Basic + Climate	0.783 (0.743–0.819)	0.694	0.818	0.65	0.896	0.493	0.896	0.818	0.856
Random Forest	Basic	0.781 (0.741–0.817)	0.68	0.808	0.663	0.911	0.449	0.911	0.808	0.856
	Basic + Climate	0.754 (0.713–0.791)	0.656	0.798	0.584	0.879	0.434	0.879	0.798	0.837
XGBoost	Basic	0.783 (0.743–0.819)	0.692	0.817	0.653	0.899	0.485	0.899	0.817	0.856
	Basic + Climate	0.783 (0.743–0.819)	0.692	0.817	0.653	0.899	0.485	0.899	0.817	0.856
Neural Network	Basic	0.781 (0.741–0.817)	0.700	0.823	0.636	0.885	0.515	0.885	0.823	0.853
	Basic + Climate	0.739 (0.698–0.778)	0.671	0.813	0.538	0.827	0.515	0.827	0.813	0.82

^1^ AUC—area under the curve, PPV—positive predictive value, and NPV—negative predictive value.

**Table 3:** Variable importance in logistic regression.

Rank	Variable	Importance ^1^
1	RSV	8.421
2	Respiratory disease	7.923
3	Age	3.478
4	Metapneumovirus	3.226
5	Average temperature	2.888
6	Neuromuscular disease	2.864
7	Average dew point	2.822
8	Bocavirus	2.061
9	Average humidity	1.792
10	SARS-CoV-2	1.748

^1^ Measured as the absolute value of the t-statistic.

**Table 4:** Verification of robustness of results of logistic models using adversarial approach.

	Original Dataset				Adversarial Dataset			
	Accuracy	Precision	Recall	F1	Accuracy	Precision	Recall	F1
Basic covariates								
Outcome = 0	-	0.82	0.89	0.86	-	0.82	0.89	0.85
Outcome = 1	-	0.65	0.51	0.57	-	0.64	0.49	0.55
Average	0.78	0.74	0.7	0.71	0.78	0.73	0.69	0.7
Weighted average		0.77	0.78	0.78		0.77	0.78	0.77
Basic + climate covariates								
Outcome = 0	-	0.83	0.9	0.86	-	0.83	0.89	0.86
Outcome = 1	-	0.67	0.53	0.59	-	0.66	0.53	0.59
Average	0.79	0.75	0.71	0.73	0.79	0.74	0.71	0.72
Weighted average		0.78	0.79	0.79		0.78	0.79	0.78

## Data Availability

Data available upon reasonable request.
